# Effect of pre-milking teat disinfection on new mastitis infection rates of dairy cows

**DOI:** 10.1186/s13620-018-0122-4

**Published:** 2018-04-18

**Authors:** David Gleeson, Jimmy Flynn, Bernadette O’ Brien

**Affiliations:** Teagasc, Animal & Grassland Research and Innovation Centre, Moorepark Co Cork, Fermoy, Ireland

**Keywords:** Teat disinfection, Somatic cell count, Dairy cows, Teat condition

## Abstract

**Background:**

The practise of teat disinfection prior to cluster attachment for milking is being adopted by farmers in Ireland, particularly where there are herd issues with new infection rates. Pre-milking teat disinfection has been shown to reduce bacterial numbers on teat skin and to be most effective against environmental bacteria such as *Escherichia coli* and *Streptococcus uberis.* A split udder design experiment was undertaken on two research herds (A = 96 cows: B = 168 cows) to test the benefit of pre-milking teat disinfection on new mastitis infection levels. The disinfectant was applied to the left front and right hind teats of all cows in each herd and the right front and left hind teats received no disinfectant treatment prior to milking over a complete lactation. Individual quarter foremilk samples were taken on 5 occasions during the lactation and all clinical cases were recorded. The presence and number of staphylococcus and streptococcus bacteria on teat skin of a random sample of experimental cows (*n* = 20) was measured on 3 occasions during lactation (April, June, and October).

**Results:**

Pre-milking teat disinfection had no significant impact on quarter SCC and new infection rates (*P* > 0.05). The median SCC was 169 (95% CI = 144–198) × 10^3^ cells/mL and 170 (95% CI = 145–199) × 10^3^ cells/mL for disinfected teats and non-disinfected teats, respectively. There were no differences in SCC observed between herds (A = 161 (95% CI = 127–205) × 10^3^ cells/mL; B = 169 (95% CI = 144–198) × 10^3^ cells/mL) over the complete lactation. Bacterial levels on teat skin were reduced significantly with pre-milking teat disinfection compared to teats receiving no disinfectant (*P* < 0.001). Total infections (clinical and sub-clinical) were similar for disinfected teats (*n* = 36) and not disinfected teats (*n* = 40), respectively. *Staphylococcus aureus* (*n* = 47) and *Strep. uberis* (*n* = 9) were identified as the predominant bacteria in quarter foremilk samples with both clinical and sub-clinical infections.

**Conclusion:**

SCC and new infection rates were similar in non-disinfected teats and disinfected (pre-milking) teats. The routine application of pre-milking teat disinfectant in pasture-grazed herds is unlikely to be of benefit where herd SCC is below 200 × 10^3^ cells/mL.

## Background

The teat orifice is an important first line of defence in protecting a cow from the invasion of mastitis pathogens into udder quarters. *Staphylococcus aureus* is one of the major and more virulent pathogens that can cause subclinical mastitis infection. Colonization of teat skin with *Staph. aureus* increases the risk of intramammary infection [1–3]. Bacterial numbers on teats prior to cluster application may be influenced by the pre-milking teat preparation procedure. A teat cleaning procedure which includes wet cleaning followed by manual drying with a paper towel will result in the lowest bacterial counts [4–7]. Pre-milking teat disinfection has been shown to reduce bacterial numbers on teat skin [8, 9]. In some studies, the concentration of *Staph. aureus* recovered by teat skin swabbing was lower when teats were dipped with an iodine disinfectant solution post-milking compared to untreated teats [10, 11]. Similarly, the use of chlorohexidine digluconate has been shown to have a significant efficacy against *Staph. aureus* [12] and *Streptococcus agalactiae* [13] under experimental challenge conditions. In particular, pre-dipping with disinfectant has been found to be most effective against environmental bacteria such as *Escherichia coli* and *Strep. uberis* [14]. In general, when cows were housed indoors the procedure was found to reduce the incidence of new intramammary infection (IMI) caused by environmental pathogens by greater than 50%. A controlled pre-milking teat disinfection study in pasture-grazed commercial dairy herds in Australia indicated no significant benefit of pre-milking disinfection when *Strep. uberis* was the most common pathogen isolated [15]. This organism is associated with the environment and is found in paddocks and roadways. The results of a controlled study in New Zealand where cows were fed outdoors on pasture, with a similar calving pattern to that in Ireland, indicated that pre-milking disinfection in addition to post-milking disinfection did not reduce incidence of new IMI for *Staph. aureus* or *Strep. uberis* [16]. In a study undertaken to profile the pathogens in clinical mastitis cases in Irish milk recording herds, staphylococcus was isolated as the predominant pathogen in 23% of samples [17]; thus, there may be reduced benefit in pre-milking teat disinfection to target contagious bacteria. The practise of pre-milking teat disinfection is being adopted by farmers in Ireland (14%) [18], particularly where there are individual farm issues with regard to raised milk SCC levels and new infection rates. While pre-milking disinfection may be considered to have a benefit in preventing the spread of infection in these situations, it may have little benefit when milk SCC is < 200 × 10^3^ cells/mL. This is the level at which bonus payments are introduced at processor level in Ireland. The effectiveness of pre-milking teat disinfection may also be dependent on the level of organic material present on teats at milking time [19]. Correct pre-milking disinfection procedure involves cleaning teats, fore-stripping, applying disinfectant product, allowing recommended contact time (15 to 30 s), drying each teat separately, before attaching clusters to a dry udder. On Irish farms, however, pre-milking teat disinfectant is generally applied directly to teats without prior cleaning, which may impact on the antimicrobial effectiveness of the disinfectant. Thus, the objective of this study was to investigate if teat disinfectant applied pre-milking, to teats not previously cleaned, would have any additional benefit when the herd SCC was < 200 × 10^3^ cells/mL.

## Methods

A split udder design experiment was undertaken (with license under the Cruelty to Animals Act, 1876 (ref B100/445)) on two Teagasc research herds (Herd A, Solohead and Herd B, Kilworth) to test the benefit of pre-milking in addition to post-milking teat disinfection on new mastitis levels. Herd A had 105 spring calving cows of which 5 were excluded from the study due to mastitis infections before trial start date, 2 cows were unsuitable due to dry teats and 2 deaths occurred during the study period, resulting in 96 cows in experimental herd A. Herd B had 253 spring calving cows of which 73 cows were assigned to a separate milking system (robotic milking), 6 cows were excluded from the study due to mastitis infections before trial start date and 2 were omitted due to excessive teat warts and 4 cow deaths occurred during the trial period, resulting in 168 cows in experimental herd B.

The mean herd parity was 3.6 and 1.6 for herds A and B, respectively. Two ready-to-use teat disinfectant products recommended for pre-milking application were applied manually using foaming cups, over a complete lactation. The two disinfectant treatment products applied to teats were Deosan teatfoam (Chlorhexidine, polyhexamethylene biguanide, Johnson Diversey) and Supercow teatfoam (Polymoric biguanide hydrochloride/Eucalyptus oil, Milk solutions Ltd.,) on Farm A and Farm B, respectively. The efficacy of both disinfectant products was previously evaluated [20]. The left front (LF) and right hind (RH) teats of all cows in each herd received pre-milking teat disinfectant (PTD). The disinfectant was applied to teats when cows were stalled for milking, without any pre-cleaning of teats. Approximately 30 s after disinfection, teats were dry wiped using disposable paper towels, before cluster attachment. On both farms, the right front (RF) and left hind (LH) teats received no pre-milking teat disinfectant treatment (NPTD) but did receive a pre-milking cleaning treatment (teats washed and dried with paper) if teats had a high hygiene score (> 30% dirt) [21] which was approximately 2% of teats at each milking occasion. Teat disinfectant was applied post-milking to all four teats using the same products used for pre-milking teat disinfection on respective farms. A high standard of cow and environmental hygiene was maintained throughout the study. Collecting yards and parlour approach yards were cleaned daily and roadways were maintained in good condition. Cow tails were clipped post calving, during mid-lactation and in late lactation. Milk liners were changed every 2000 cow milkings. Both herds were managed outdoors and offered grass within two weeks of calving and remained outdoors until November when housed in cubicles and offered grass silage.

### Quarter milk sampling procedure

Individual quarter foremilk milk samples were taken in an aseptic manner on 5 occasions during a complete lactation: post-calving (7 to 14 days post calving, sample 1), May (average DIM =75, sample 2), July (average DIM =131, sample 3), August (average DIM =194, sample 4), October (average DIM =242, sample 5). Mean calving date for both herds was 20th of February. Samples were examined using the International Dairy Federation guidelines for microbiological analysis [22]. SCC was assessed using a Fossomatic FC (Foss Electric, Hillerød, Denmark). Quarter foremilk samples collected post-calving with a SCC > 500 × 10^3^ cells/mL, and/or quarters treated for clinical mastitis prior to allocation to treatment, were excluded from the data set. Quarters were considered clinically infected if the milk was visibly abnormal or if quarters had signs of inflammation. When an individual quarter SCC was > 500 × 10^3^ cells/ mL and pathogens were isolated the quarter was considered to have a new sub-clinical infection. Sub-clinical infections which subsequently became clinical were excluded from the sub-clinical data set.

### Teat swabbing procedure

To establish the presence and number of staphylococcus and streptococcus bacteria on teat skin, a random sample of cows (*n* = 20) were selected on 4 occasions during lactation (April, June, August, and October) on both farms. All teats from the selected cows were swabbed using two sterile swabs (Cultiplast, LP Italian SPA, Via Carlo Reale, 15/4, 20,157, Milano, Italy); one swab was used for the two teats receiving no disinfectant (RF & LH) and another swab was used for the two teats that received disinfectant (LF & RH). Teats were swabbed before the application of disinfectant (in the treated quarters) and before cluster attachment for milking. Swabs were drawn across the teat orifice and down the side of each teat avoiding contact with the udder hair. Immediately after swabbing was completed, the swabs were placed in individual sterile bottles containing 5 mL Trypticase Soy Broth. The broth was prepared in 500 mL amounts and autoclaved at 121 °C for 15 min, and then distributed into 5 mL aliquots in a Laminar Flow Cabinet. The sterile bottles containing the swabs were frozen (-20 °C) until analysed.

### Identification of bacteria

The swabs were subsequently plated on two separate selective agars: Baird-Parker (staphylococcus) and Edwards (streptococcus). Specific bacteria types within each category were not defined. Following incubation at 35–37 °C for 48 h, colony counts (cfu/mL) on agar plates for each pathogen type were manually counted. Plates with numerous (NS) colony counts were assigned a count of at least 45 (cfu/mL) and plates with infinite (IF) colony counts were assigned a count of at least 100 (cfu/mL) to accommodate numeration and for statistical analysis.

### Teat hyperkeratosis

Teat orifices were classified for hyperkeratosis (HK) using a severity scale of 1 to 5 [23]. Score 1 was a normal teat-end orifice; Score 2 was a slight smooth or broken ring of keratin; Score 3 was a moderate raised smooth or broken ring of keratin; Score 4 was a large raised smooth or broken ring of keratin; Score 5 was a severe broken ring of keratin. All teat inspections were conducted by the same observer and were carried out on 3 occasions coinciding with quarter sampling dates in May, July and October. The operator, using a lamp to illuminate the teat-ends, classified all teats for HK immediately after cluster removal at the morning milking. A total HK score for each cow on each inspection was obtained by calculating the average score of the four teats. The average score for disinfected (LF, RH) and non-disinfected teats (RF, LH) was calculated by averaging the score for each set of teats.

### Statistical analysis

Statistical analysis of data was performed using SAS software (SAS 2009) [24]. The statistical analysis was performed using Proc Glimmix with cow as a random effect. Farm, treatment, teat position and sample day were the fixed effects, along with cow lactation number and calving date as covariates. These variables were analysed using the following model:

LogSCC = μ (mean) + Farm + Sample No. + Treatment + Sample No.* Treatment + Lactation No. + Calving date + e (residual error).

Where there were repeated measures over time this was modelled using covariance structures. Comparisons were made for SCC between farms, treatments, teat position, sampling day, cow lactation number and for interactions between sampling day and teat treatment. Interactions for covariates were tested and not included in the final model when not significant. Somatic cell counts were averaged across teats within treatment (LF & RH versus RF & LH) and for teat position (RF versus LF & RH versus LH) across treatments. Somatic cell counts were rescaled by dividing by a factor of 10 before log transformation for distributional reasons. Results are presented as back transformed median data.

Teat hyperkeratosis score was analysed for farm, sample time, treatment and for the interaction for time and treatment. These variables were analysed using the following model:

Teat score = μ (intercept) + Farm + Time + Treatment + Time* Treatment + e (residual error). The bacterial counts on teat skin were analysed directly but large numbers were not easily measured and were recorded as either category NS or IF. Plates with numerous (NS) colony counts were assigned a count of at least 45 (cfu/mL) and plates with infinite (IF) colony counts were assigned a count of at least 100 (cfu/mL). The lower limit on these counts was treated as a censoring level (e.g., the outcome was measured but known only to be at least 100) and censored regressions were fitted with Proc NLMIXED. Differences in bacterial numbers observed on cow’s teats were analysed by categorising results and using Fisher’s exact test. The interaction between treatment (pre-milking disinfection and no pre-milking disinfection) and sampling day (April, June, and October) were included in the analysis. The results are presented as the average microbial counts (cfu/mL) over 3 sampling dates, for bacteria type (staphylococcus and streptococcus), on farms (A & B) and for teat disinfection treatments.

Two by two tables including bacteria type and treatment and categories of somatic cell counts (< 100 × 10^3^, 101–200 × 10^3^, 201–300 × 10^3,^ 301–400 × 10^3^ and > 401 × 10^3^ cells/mL) were tested using chi-square statistics.

## Results

**Fig. 1:**
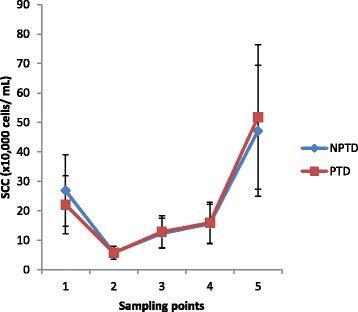
Median somatic cell count from two herds and for two pre-milking teat disinfection treatments. Median somatic cell count (× 10^3^ cells/mL) from two herds (A, B) and for two pre-milking teat disinfection treatments (PTD: that received pre-milking teat disinfectant and NPTD: did not receive disinfectant) at five sampling points during lactation. Sample points: 1 = post-calving (Feb/March), 2 = May, 3 = July, 4 = August, 5 = October. Error bars show the 95% confidence intervals

There were no significant differences in the median SCC observed in quarter foremilk samples between herds (A = 161 (95% CI = 127–205) × 10^3^ cells/mL: B = 169 (95% CI = 144–198) × 10^3^ cells/mL) over the complete lactation (*P* > 0.05) (Table 1). The median SCC was 169 (95% CI = 144–198) × 10^3^ cells/mL and 170 (95% CI =145–199) × 10^3^ cells/mL for PTD teats (LF + RH) and NPTD teats (RF + LH), respectively, over the complete lactation. There was no interaction between pre-milking treatment and herd. There was a significant interaction between teat position and sampling day (P = < 0.01). Milk samples from the combined front teats (174 (95% CI = 145–208) × 10^3^ cells/mL) had higher SCC than milk from the combined hind teats (156 (95% CI = 130–187) × 10^3^ cells/mL) (*P* < 0.05), regardless of treatment applied or farm location. Milk samples from individual front teats (RF v LF) were compared for SCC and likewise when individual hind teats (RH v LH) were compared, there were no significant differences in SCC observed (*P* > 0.05). There was no interaction between teat disinfectant treatment and sampling day (P > 0.05) (Table 1). The combined median SCC from herds A and B for PTD and NPTD treatments at each sample test day are presented in Fig. 1. Somatic cell count differed between sampling days (*P* < 0.001) (Fig. 1), with sample day 5 significantly different from all other sample days (*P* < 0.05). The highest SCC observed for sample 5 corresponded with the latter part of the milk production season. The proportion of quarter foremilk samples with an SCC (cells/mL) within categories < 100 × 10^3^, 101–200 × 10^3^, 201–300 × 10^3^, 301–400 × 10^3^ and > 401 × 10^3^ cells/mL, across 5 sampling dates are presented in Table 2. The proportion of quarters with SCC < 100 × 10^3^ cells/mL for herd A (range = 0.77 to 0.90) and herd B (range = 0.81 to 0.92) and with an SCC > 401 × 10^3^ cells/mL (A range = 0.05 to 0.07: B range = 0.02 to 0.07), were similar for disinfectant treatments within herds and across herd for sampling dates 1, 2, 3 and 4. However, the proportion of quarters with an SCC > 401 × 10^3^ cells/mL and less than ≤200 × 10^3^ increased and decreased proportionally by 0.12 and 0.21, respectively for herd A compared to herd B at the 5th sampling date (Table 2). The number of clinical cases observed throughout the lactation tended to be lower with PTD teats (*n* = 18) compared to NPTD teats (*n* = 26) (Table 3). However, the number of sub-clinical cases was higher for PTD quarters (n = 18) compared to NPTD quarters (*n* = 14). Therefore, total infections (clinical and sub-clinical) were similar for disinfected teats (*n* = 36) and not disinfected teats (*n* = 40), respectively. Clinical cases of mastitis on herd A tended to occur post calving (*n* = 12), whereas the highest number of cases occurred during the July period (n = 12) for herd B. Sub-clinical cases were highest for both herds during the late lactation period (October).

**Table 1:** Median somatic cell count (× 10^3^ cells/mL) for two herds (A, B) and for two pre-milking teat disinfection treatments (PTD: that received pre-milking teat disinfectant and NPTD: did not receive disinfectant) at five sampling dates during lactation

Herd	A (*n* = 97)	B (*n* = 167)	*p*-value			
	161 (127–205)	169 (144–198)	0.54			
Treatment	PDT	NPDT				
	169 (144–198)	170 (145–199)	0.93			
Herd	A			B		
Sample day	PTD	NPTD	*p*-value	PTD	NPTD	*p*-value
1-post- calving	98 (68–141)	125 (87–180)	0.98	352 (272–456)	409 (316–530)	0.99
2-May	34 (23–50)	31 (21–46)	1.00	75 (58–97)	80 (62–104)	1.00
3-July	156 (109–223)	119 (83–171)	0.97	120 (93–156)	134 (104–174)	0.99
4-August	231 (160–333)	279 (194–402)	0.99	134 (103–173)	115 (88–148)	0.99
5-October	1300 (905–1877)	1411 (981–2025)	1.00	301 (230–392)	246 (189–321)	0.96

() parentheses represent a 95% confidence interval

Total observations across sample dates = 5075

**Table 2:** Proportion of quarter foremilk samples from two herds (A, B) and for two pre-milking teat disinfection treatments (PTD: that received pre-milking teat disinfectant and NPTD: did not receive disinfectant) with a somatic cell count (SCC), < 100 × 10^3^, 101–200 × 10^3^, 201–300 × 10^3,^ 301–400 × 10^3^, and > 401 × 10^3^ cells/mL, across 5 sample dates

		PTD		NPTD	
Sample day	SCC × 10^3^ cells/mL	A	B	A	B
1		*n* = 185	*n* = 325	*n* = 179	*n* = 328
	0–100	0.89	0.83	0.87	0.81
	101–200	0.03	0.06	0.07	0.07
	201–300	0.02	0.02	0.01	0.02
	301–400	0.01	0.02	0.01	0.03
	401+	0.05	0.07	0.04	0.06
2		*n* = 160	*n* = 330	*n* = 157	*n* = 329
	0–100	0.90	0.90	0.91	0.90
	101–200	0.04	0.04	0.03	0.05
	201–300	0.01	0.03	0.01	0.01
	301–400	0.01	0.01	0.01	0.00
	401+	0.04	0.02	0.04	0.03
3		*n* = 192	*n* = 334	*n* = 193	n = 334
	0–100	0.85	0.92	0.88	0.91
	101–200	0.03	0.02	0.03	0.03
	201–300	0.02	0.01	0.03	0.01
	301–400	0.02	0.01	0.01	0.01
	401+	0.07	0.04	0.06	0.04
4		*n* = 187	*n* = 331	*n* = 186	*n* = 327
	0–100	0.81	0.89	0.77	0.90
	101–200	0.08	0.05	0.08	0.05
	201–300	0.03	0.01	0.08	0.02
	301–400	0.01	0.00	0.01	0.01
	401+	0.07	0.05	0.06	0.03
5		n = 193	*n* = 308	*n* = 190	*n* = 307
	0–100	0.46	0.79	0.48	0.84
	101–200	0.21	0.10	0.24	0.07
	201–300	0.09	0.02	0.05	0.02
	301–400	0.07	0.02	0.04	0.02
	401+	0.17	0.07	0.19	0.05

Differences in quarter foremilk sample numbers within herds and between sample days are due to non-collected or unread quarter foremilk samples

**Table 3:** New mastitis infection rates from two herds (A, B) and for two pre-milking teat disinfection treatments (PTD: that received pre-milking teat disinfectant and NPTD: did not receive disinfectant) and bacterial species identified from those infections

Herd	A		B	
	PTD	NPTD	PTD	NPTD
Clinical	10	12	8	14
†Sub-clinical	9	6	9	8
Total infections	19	18	17	22
	Clinical		Subclinical	
Bacteria	PTD	NPTD	PTD	NPTD
Not detected	9	10	0	0
*Staphylococcus aureus*	6	15	16	10
*Streptococcus uberis*	3	1	2	3
*Streptococcus dysgalactiae*	0	0	0	1

† Quarter milk samples with an SCC ≥500 × 10^3^ cells/mL with a pathogen present

*Staph. aureus* (*n* = 47) and *Strep. uberis* (*n* = 9) were identified as the predominant bacteria in quarter foremilk samples with both clinical and sub-clinical infections. A small number (n = 3) of *Staph. aureus* pathogens were identified as non-haemolytic.

Staphylococcus and streptococcus bacterial counts on teat skin were significantly higher for herd A compared to herd B. (*P* < 0.001)(Table 4). Both staphylococcus and streptococcus bacterial counts were significantly lower on disinfected teats compared to non-disinfected teats prior to cluster application (P < 0.001) (Table 4). There was no treatment by day interaction for both bacteria types (*P* > 0.05).

**Table 4:** Total *Staphylococcus* and *Streptococcus* bacterial counts (cfu/mL) on teat skin from two herds (A, B) and for two pre-milking teat disinfection treatments (PTD: that received pre-milking teat disinfectant and NPTD: did not receive disinfectant) across four sampling points during lactation

	Herd A	Herd B	s.e.	Significance *p*-value
Staphylococcus (cfu/mL)	52.4	14.5	3.76	< 0.001
Streptococcus (cfu/mL)	27.2	13.5	3.48	< 0.001
	NPTD	PTD		
Staphylococcus (cfu/mL)	50.1	16.8	3.76	< 0.001
Streptococcus (cfu/mL)	30.1	10.6	3.47	< 0.001

cfu/mL = Colony forming units per millilitre

Total number of observations = 612

Teat hyperkeratosis score was significantly higher for herd A (2.03) compared to herd B (1.64) (P < 0.001) and increased with stage of sampling (P < 0.001), which coincided with lactation stage (Table 5). There were no differences observed in the hyperkeratosis score for teats disinfected compared to those not disinfected prior to milking (P > 0.05). Furthermore, there was no sampling stage by treatment interaction (P > 0.05).

**Table 5:** Mean teat hyperkeratosis score (HK) for each herd (A, B), and for two pre-milking teat disinfection treatments (PTD: that received pre-milking teat disinfectant and NPTD: did not receive disinfectant) and at three sampling points during lactation

	HK	s.e.	Significance *p*-value
Herd A	2.04		
Herd B	1.64	0.04	< 0.001
PTD	1.83		
NPTD	1.84	0.05	0.82
Day 1 (April)	1.73		
Day 2 (July)	1.81		
Day 3 (October)	1.98	0.03	< 0.001

Not significant for treatment * time

No of observations = 1602

## Discussion

This study was undertaken to measure the impact of teat disinfection prior to cluster attachment for milking, on quarter milk sample SCC, new infection rates, teat-end condition and bacterial counts on teat skin. Previous international studies used herd comparison with commercial herds [15, 16, 25] to measure the impact of pre-milking teat disinfection. In this study, a split udder design experiment was used on two research herds. Pre-milking teat disinfection had no impact on individual quarter SCC.

A review on mastitis control measures suggested SCC was more related to milking procedures such as wearing gloves during milking, using postmilking teat dipping, and yearly inspection of milking equipment rather than pre-dipping which did not have a statistically significant impact on bulk milk tank SCC [8]. The proportion of quarters with an SCC above the European Council Directive 92/46/EEC limit of 400 × 10^3^ cells/mL for bulk tank milk or the proportion of quarters with an SCC < 200 × 10^3^ cells/mL did not differ between pre-milking teat treatment. The increase in the proportion of quarters > 400 × 10^3^ cells/mL with herd A compared to herd B towards the end of lactation may be more related to farm management practises on individual farms when cows were indoors [26]. The increase in quarter SCC observed with lactation stage (Fig. 1) was expected as cow milk yields decrease and infection levels increase [27]. Overall geometric herd bulk milk SCC supplied by the milk processor for early lactation (March), for herds A and B were 126 × 10^3^ cells/mL and 141 × 10^3^ cells/mL, respectively, indicating a low initial infection level for both herds at the beginning of the lactation. The low initial herd SCC and the small difference in new intramammary infections observed may account for no significant differences in SCC levels observed over the lactation for milk from disinfected teats compared to milk from teats that did not receive pre-milking teat disinfectant.

The lack of evidence of benefit in pre-milking teat disinfection as measured by the number of clinical cases is in agreement with a number of studies where herds were managed under similar conditions. The authors of an Australian study concluded that pre-milking disinfection is unlikely to reduce clinical mastitis incidence or new infection rates when compared to no teat preparation in commercial herds. The authors also concluded that there may be benefits of pre-milking teat disinfection, if teats were heavily soiled when presented for milking [15]. Similarly, in a New Zealand study where herds were randomly allocated to pre-milking disinfectant treatments post calving, no reduction in the incidence of new IMI for any pathogens including *Staph. aureus* and *Strep. uberis* were observed [16]. The majority of clinical and sub-clinical infections in this present study were associated with *Staph. aureus* followed by *Strep. uberis*. While there may be benefits in terms of milk let down with pre-milking disinfection, the cost benefit of using a disinfectant was considered not justified when compared to good teat preparation (wash and dry with paper) [28]. Increased levels of supervision for the research herds and a high standard of parlour, environment and cow hygiene, in addition to both herds having a low average parity (2.3 lactations), may have contributed to the low infection levels for the non-disinfected teats. Lower SCC would be expected from herds with lower parity [29] and herds with better hygiene [26]. Using a split udder design experiment may also have impacted on the trial results as 9 cows (6 herd A, 3 herd B) had clinical infections in both disinfected and in non-disinfected quarters. This may be due to cross infection at milking time. This trend was not observed for sub-clinical infections.

Both staphylococcus and streptococcus bacterial counts were lower on teat skin when teats were disinfected and dried with paper compared to teats with no preparation, prior to cluster attachment. These results agree with previous studies which showed that teat disinfection reduced bacterial levels on teat skin [9, 30]. However, the reduction in bacterial counts on teat skin did not result in lower mastitis infection levels, even though *Staph. aureus* was the predominant pathogen identified in cultured sub-clinical milk samples*.* Higher bacterial counts were observed on teats from herd A compared to herd B. The teat disinfectant products used both for pre and post milking on the different farms may partially account for differences in bacterial counts observed on teat skin. However, differences in environmental conditions and milking management are more likely to have accounted for these differences as bacterial levels on teat skin were lower on herd B regardless of the pre-milking teat disinfection treatments applied.

Teat hyperkeratosis score did not differ for teats disinfected as compared to those not disinfected pre-milking. Hyperkeratosis would be expected to increase with lactation stage with the lowest levels observed at calving and increasing up to 120 days post calving and remaining static thereafter [31]. The higher teat score observed with herd A may be related to differences in teat disinfectant emollient properties, milk machine settings, environment or that the herd had a higher average lactation number (3.6) compared to herd B (1.6), as teat score tends to increase with lactation number [23].

## Conclusion

From a mastitis infection point of view there was no benefit observed in applying teat disinfectant prior to cluster attachment. The low initial herd SCC combined with a low herd parity, a high standard of parlour and environment hygiene, post-milking teat disinfection, regular liner changes, and regular cow tail clipping may all have contributed to maintaining low infection levels in the non-disinfected teats. Furthermore, the application of disinfectant to un-cleaned teats may have impacted on the effectiveness of the disinfectant products. In situations where herd infection levels are considered high and where the risk of spread of infection is greater, then there may be benefit in pre-milking teat disinfection of clean teats, followed by teat drying. However, the routine application of pre-milking teat disinfectant in pasture-grazed herds is unlikely to be of benefit where herd SCC is below 200 × 10^3^ cells/mL.
